# Prolonged screen time is associated with increased prevalence of sleep disorders in children

**DOI:** 10.1007/s00431-026-07284-9

**Published:** 2026-08-06

**Authors:** Bruna Luiza Maximo Ramos, Rafaella Bom dos Santos Hochuli, José Vitor Nogara Borges de Menezes, Juliana Feltrin-Souza

**Affiliations:** Department of Stomatology, Federal University of Paraná, Av. Prefeito Lothário Meissner, 632 - Jardim Botânico, Curitiba, Paraná, 80210-170 Brazil

**Keywords:** Sleep–wake transition disorders, Screen time, Child, Preschool, Sleep

## Abstract

**Purpose:**

This study aimed to investigate whether sleep disorders are associated with electronic screen use before bedtime and total daily usage time in preschool children.

**Methods:**

A cross-sectional study was conducted with 604 preschool children aged 4–5 years. Sleep disorders were assessed using the Sleep Disturbance Scale for Children (SDSC). Caregivers reported children’s daily screen time and use of electronic media at bedtime. Data were analyzed using univariate and multivariate Poisson regression models with robust variance (p<0.05).

**Results:**

Most children used electronic media to fall asleep (88.9%), and more than half used it for over one hour per day (66.9%), while 17.3% used it for more than 4 hours daily. Prolonged electronic screen time was independently associated with higher SDSC scores (p = 0.003; adjusted PR = 1.16; 95% CI = 1.05 – 1.28), as well as with the excessive daytime sleepiness (p = 0.015; adjusted PR = 1.16; 95% CI = 1.05 –1.28) and sleep-wake transition disorders (p = 0.026; adjusted PR = 1.12; 95% CI = 1.01 – 1.25). Use of media at bedtime was associated with disorders of initiating and maintaining sleep (p = 0.010; adjusted PR = 1.17; 95% CI = 1.04 – 1.33) and hyperhidrosis (p = 0.046; adjusted PR = 1.12; 95% CI = 1.00 – 1.27).

*Conclusion*: Prolonged screen time and screen use before bedtime are associated with sleep disorders in preschool children.

**Table:** 

**What is known:** • *Excessive and problematic screen use has been widely reported among children and adolescents,raising concerns about its potential impact on sleep health and children development.* **What is New:** • *Higher screen exposure was independently associated with sleep disturbances in preschool children, emphasizing early childhood as a critical window for interventions aimed at reducing screen-related sleep problems.*

## Introduction

Sleep is vital for children’s physical and mental growth and development [1, 2]. Several factors can negatively influence sleep, and recent studies have demonstrated an association between prolonged use of electronic media and poorer sleep quality [3–7]. Due to the growing digital market targeting children, concerns have increased regarding excessive screen exposure, particularly among young children (0–5 years), and its potential harmful effects [8, 9].

The American Academy of Pediatrics (AAP) has established recommendations for media use among children and adolescents with the objective of enhancing their quality of life [7, 10]. According to these guidelines, as well as those from the Brazilian Society of Pediatrics (BSP), screen time for children aged 2 to 5 years should be limited to a maximum of 1 h per day, and exposure should be avoided within 1 to 2 h before bedtime [10, 11].

In this context, sleep disturbances in early childhood have emerged as an important public health concern, as accumulating evidence suggests that poor sleep is associated with emotional and behavioral problems, deficits in attention, learning, and self-regulation, impaired neurocognitive development, increased risk of excess body weight, and adverse cardiometabolic outcomes [6, 12, 13, 14, 15, 16]. Because the preschool period represents a critical window for brain development and behavioral regulation, inadequate sleep may negatively affect both immediate health and long-term developmental trajectories [6, 12, 13, 14, 15, 16]. In addition, sleep bruxism is frequently associated with sleep disorders [17] and is commonly reported in preschool-aged children, potentially contributing to tooth wear, orofacial discomfort, and headaches [18]. It has been associated with early signs of temporomandibular disorders.

Despite growing evidence on the association between screen time and sleep outcomes in children and adolescents, studies specifically focusing on preschool-aged children remain limited, particularly in middle-income countries [6]. Based on the hypothesis that prolonged daily screen time and media use before bedtime may be associated with a higher prevalence of sleep disturbances, this study aimed to assess the prevalence of sleep disorders and test their association with screen time exposure in preschool-aged children. Additionally, the study examined potential associations between media use patterns and sleep disorders.

## Materials and methods

A population-based cross-sectional study was conducted between February and December 2018. The study involved preschool children aged 4 and 5 years enrolled in public schools in the municipality of Itajaí, Santa Catarina, Brazil. Itajaí is a coastal city in southern Brazil, with an estimated population of 287.289 inhabitants and a Human Development Index (HDI) of 0.795, classified as high [19]. The study protocol was approved by the Ethics and Research Committee of the Federal University of Paraná (approval number 2.646.765). Written informed consent was obtained from parents or legal guardians prior to participation. The study was reported according to STROBE guidelines.

The inclusion criteria comprised children aged 4 to 5 years who were regularly enrolled in municipal public schools in Itajaí and whose parents or legal guardians provided informed consent for participation. Children with diagnosed syndromes or with incomplete questionnaires were excluded from the study.

The sample size was calculated for an infinite population, assuming a 50% prevalence of sleep disorders among preschool-aged children, a 95% confidence level, and a 5% margin of error. A design effect of 1.4 was applied to account for cluster sampling, resulting in a minimum required sample size of 538 participants. After adding 20% for potential losses, the final target sample size was 645 children. A probabilistic cluster sampling strategy was used, as previously described in a study involving the same population [20].

A structured questionnaire, specifically developed for this study, was distributed to parents or legal guardians. It included socioeconomic variables (household income, educational attainment, and marital status) and demographic characteristics (age and sex of the child and caregivers). Screen time exposure was referred to domestic exposition rather than specific patterns of screen use. It was assessed using two questions. The first addressed daily screen time: “How many hours per day does your child use devices such as cell phones, tablets, or television?”, with response options of up to 30 min, 30 min to 1 h, 1–2 h, 2–3 h, and more than 4 h. The second question assessed media use at bedtime: “In the past 6 months, has your child used devices such as cell phones, tablets, or television to fall asleep?”, with response options of yes, no, or do not know/cannot remember.

Sleep disorders were assessed using the SDSC, a validated instrument for individuals aged 3–18 years that was translated and culturally adapted into Brazilian Portuguese [21, 22]. The SDSC is a structured questionnaire completed by parents or legal guardians, addressing sleep-related behaviors and events observed in the child over the preceding six months [21, 22].

The SDSC consists of 26 items covering six domains: disorders of initiating and maintaining sleep (DIMS), sleep breathing disorders (SBD), disorders of arousal (DA), sleep–wake transition disorders (SWTD), excessive daytime sleepiness (EDS), and sleep hyperhidrosis (SHY). Each item is scored on a 5-point Likert scale ranging from 1 (never) to 5 (daily). Total scores for each domain were categorized as absent of disorder, risk of sleep disorder, or presence of disorder, according to the cut-off points established by the authors [21, 22]. For the purposes of this study, scores were dichotomized into presence or absence of sleep disorders.

A pilot study was conducted with parents or legal guardians of 15 children to evaluate the clarity and comprehensibility of the questionnaires. The aim was to identify potential difficulties in item interpretation and detect possible ambiguities. The findings indicated good acceptability and comprehension of the instruments, and no modifications were required.

Data were statistically analyzed using SPSS 17 (IBM, USA) and STATA 12.0 (StataCorp, College Station, PA, USA). The internal consistency of the Sleep Disturbance Scale for Children (SDSC) was assessed in the present sample using Cronbach’s alpha coefficient. The 26-item scale demonstrated good internal consistency, with a Cronbach’s alpha of 0.823 (0.830 based on standardized items), indicating satisfactory reliability for assessing sleep disturbances in the study population.

The study’s outcome variable was the presence of sleep disorders in preschoolers, as assessed by the SDSC. Disorders identified in the domains DIMS, SBD, DA, SWTD, EDS, and SHY were dichotomized into pathological sleep disorders (PSD) and no sleep disorders (NSD), based on the cut-off scores recommended by the authors [17, 18]. The PSD category included only cases classified as having the disorder, while the NSD category included cases such as DIMS < 10, SBD < 3, DA < 3, SWTD < 8, EDS < 2, SHY < 7 [17, 18].

Independent variables were dichotomized based on theoretical references or the sample median. These included monthly household income (based on the study’s median), child’s gender, family structure, and caregiver’s education level. Family structure was categorized as nuclear when parents were in a stable relationship, and non-nuclear when parents reported being single, divorced, or widowed. Caregiver education was dichotomized as more than or less than eight years of schooling. Per capita income was also classified according to the median, with categories of R$ 550.00 or less and R$ 551.00 or more.

Screen time exposure was evaluated as follows: use at bedtime (yes/no), total daily screen time (less than 1 h; 1 h or more), and prolonged use (less than 4 h; 4 h or more). The categorization criteria were based on recommendations from the American Academy of Pediatrics (AAP) and the Brazilian Society of Pediatrics (BSP) [10, 11].

The association between the media use with sleep disorders was analyzed using univariate and multivariate Poisson regression with robust variance, yielding crude prevalence ratios (PR_c_) and adjusted prevalence ratios (PR_a_). Variables with *p* < 0.20 in the univariate analysis were included in the multivariate models. A stepwise approach was applied, retaining variables that remained statistically significant or contributed to model adjustment. A significance level of 5% was adopted.

Collinearity among screen time variables was assessed, including electronic media use at bedtime, daily screen time (< 1 h), and prolonged use (≥ 4 h). The variable with the best model fit was retained.

## Results

**Fig. 1:**
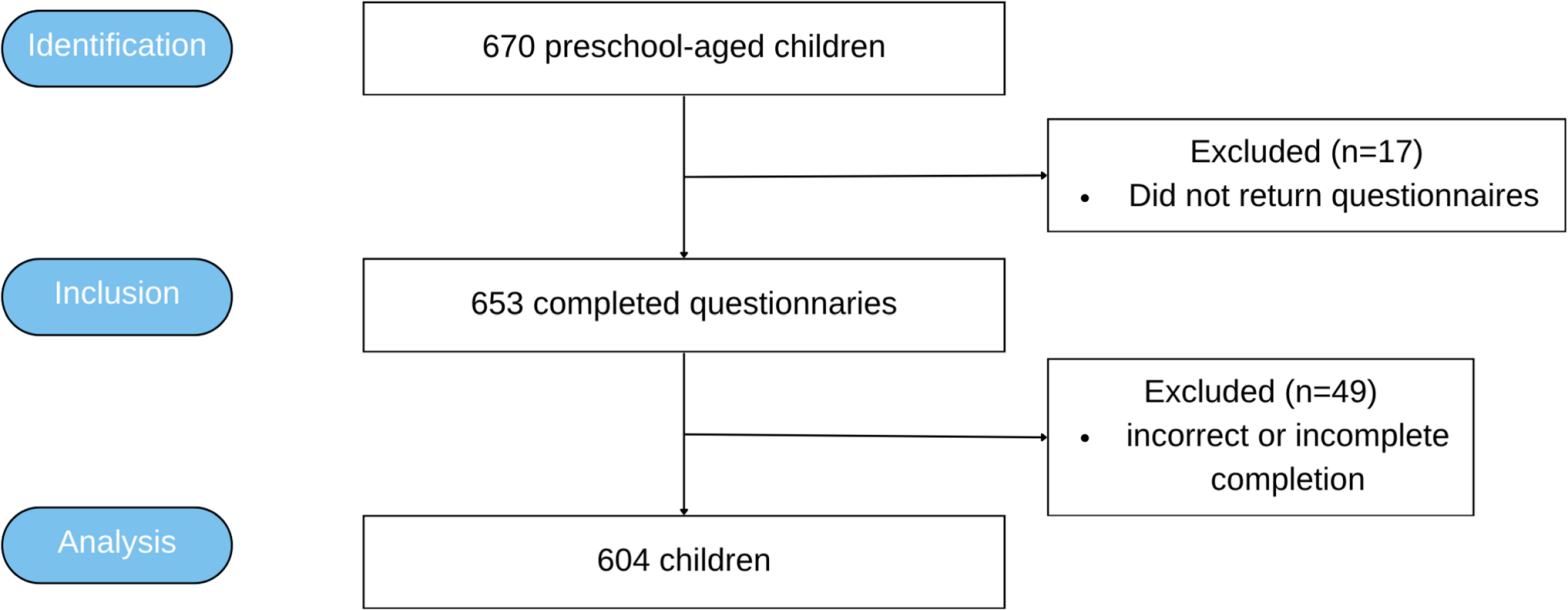
Flow of preschool-aged children participating in the study

A total of 670 preschool-aged children were invited to participate in the study. This number exceeded the initially calculated sample size to ensure proportional representation across clusters (school classes), achieved through the distribution of consent forms to parents or legal guardians. The response rate for this study was 97.4%, with 653 completed questionnaires returned. The flowchart of participants is shown in Fig. 1. Sociodemographic and economic characteristics of the study population are presented in Table 1. Sample sizes varied across variables because participants were not required to answer all questionnaire items. Missing responses were treated as missing data and excluded from the corresponding analyses.

**Table 1:** Frequency distribution of sociodemographic and economic characteristics of the study population (*N* = 604; Itajaí-SC, Brazil – 2019)

Variables	Frequency N(%)
**Gender**	
Female	322 (53.3)
Male	281 (46.5)
Not informed	1 (0.2)
**Ethnicity**	
White	434 (71.9)
Black	34 (5.6)
Asian	4 (0.7)
Indigenous	1 (0.2)
Brown	102 (16.9)
Not informed	29 (4.8)
**Guardian's education**	
More than 8 years	471 (78.0)
Less than 8 years	133 (22.0)
**Monthly family income**	
Up to R$2200.00	373 (61.8)
More than R$2200.00	231 (38.2)
**Family structure**	
Nuclear family	471 (78.0)
Non-nuclear family	133 (22.0)
**Educational centers**	
Cordeiros	152 (25.2)
Fazenda	31 (5.1)
São Vicente	67 (11.1)
São Judas	62 (10.3)
Cidade Nova	104 (17.2)
São João	67 (11.1)
Salseiros	71 (11.8)
Itaipava	50 (8.3)
**How many hours per day does the child use devices such as cell phones, tablets, television, and computers?**	
Up to 30 min	63 (11.0)
Between 30 min and 1 h	126 (22.1)
Between 1 and 2 h	155 (27.1)
Between 2 and 3 h	128 (22.4)
More than 4 h	99 (17.3)
**In the last 6 months, has the child used devices such as cell phones, tablets, television, and computers to fall asleep?**	
Yes	520 (88.9)
No	65 (11.1)

The SDSC showed good internal consistency in the study sample. Reliability analysis yielded a Cronbach’s alpha coefficient of 0.823 for the 26-item scale (0.830 based on standardized items), indicating satisfactory internal consistency and supporting the reliability of the instrument in this population.

The prevalence of media use at bedtime was 88.9% (*n* = 520), while 382 children (66.9%) reported using media more than one hour per day. Additionally, 17.3% (*n* = 99) were classified as engaging in prolonged media use (Table 1). The prevalence rates for disorders of initiating and maintaining sleep, sleep-related breathing disorders, arousal disorders, sleep–wake transition disorders, sleep hyperhidrosis, and excessive sleepiness disorders respectively are shown in Table 2.

**Table 2:** Distribution of sleep disorders in the population

Sleep disorders	Not identified *N* (%)	Risk identified *N* (%)	Disorder identified *N* (%)	Total *N* (%)
Disorders of initiating and maintaining sleep*^1^	296 (49.0)	244 (40.4)	64 (10.6)	604 (100)
Sleep-related breathing disorders*^2^	302 (50.0)	175 (29.0)	127 (21.0)	604 (100)
Disorders of arousal *^3^	400 (66.2)	143 (23.7)	61 (10.1)	604 (100)
Sleep–wake transition disorders*^4^	282 (46.75)	207 (34.3)	115 (19.0)	604 (100)
Sleep hyperhidrosis*^5^	367 (60.8)	156 (25.8)	81 (13.4)	604 (100)
Excessive sleepiness disorders*⁶	424 (70.2)	138 (22.8)	42 (7.0)	604 (100)

^*^1 scores: not identified ≤ 10; risk identified ≥ 11 and ≤ 16; disorder present ≥ 17

^*^2 scores: not identified ≤ 3; risk identified ≥ 4 and ≤ 6; disorder present ≥ 7

^*^3 scores: not identified ≤ 3; risk identified ≥ 4 and ≤ 5; disorder present ≥ 6

^*^4 scores: not identified ≤ 8; risk identified ≥ 9 and ≤ 13; disorder present ≥ 14

^*^5 scores: not identified ≤ 2; risk identified ≥ 3 and ≤ 6; disorder present ≥ 7

^*^6 scores: not identified ≤ 7; risk identified ≥ 8 and ≤ 12; disorder present ≥ 13

Regarding the association between electronic media use and sleep disturbances, prolonged use (≥ 4 h) was significantly associated with higher total SDSC scores, as well as with excessive daytime sleepiness disorder, arousal disorders, and sleep–wake transition disorders (Table 3). Daily media use of 1 h or more was also associated with higher prevalence of excessive daytime sleepiness in 16% and the total SDSC score in 10% (Table 3). Additionally, media use at bedtime was significantly associated with higher prevalence of initiating and maintaining sleep disorders (Table 3).

**Table 3:** Association of sleep disorders with the variables analyzed in the study population (*N* = 604; Itajaí-SC, Brazil – 2019)

Variables		SDSC total		Total *N*(%)	*p*-value	PR_c_ (CI 95%)
		Yes *N*(%)	No *N*(%)			
**SDSC TOTAL**						
Use before bedtime	Yes	321 (61.7%)	199 (38.3%)	520 (100%)	0.352	1.11 (0.88–1.40) 1
	No	36 (55.4%)	29 (44.6%)	65 (100%)		
Total daily screen time	> 1 h	262 (63.1%)	153 (36.9%)	415 (100%)	0.025	1.10 (1.01–1.19) 1
	< 1 h	101 (53.4%)	88 (46.6%)	189 (100%)		
Prolonged screen use	> 4 h	73 (73.7%)	26 (26.3%)	99 (100%)	0.003	1.16 (1.05–1.28) 1
	< 4 h	290 (57.4%)	215 (42.6%)	505 (100%)		
Family structure	Non-nucleated	81 (60.9%)	52 (39.1%)	133 (100%)	0.829	1.01 (0.87–1.18) 1
	Nucleated	282 (59.9%)	189 (40.1%)	471 (100%)		
Caregiver’s education	< 8 years	68 (51.1%)	65 (48.9%)	133 (100%)	0.027	0.81 (0.68–0.97)
	> 8 years	295 (62.6%)	176 (37.4%)	471 (100%)		
Per capta income (R$)	≥ 551.00	158 (67.8%)	75 (32.2%)	233 (100%)	0.002	1.26 (1.08–1.46) 1
	≤ 550.00	128 (53.8%)	110 (46.2%)	238 (100%)		
Gender	Male	171 (60.9%)	110 (39.1%)	281 (100%)	0.759	1.021 (0.89–1.16) 1
	Female	192 (59.6%)	130 (40.4%)	322 (100%)		
**HYPHERHIDROSIS**						
Use before bedtime	Yes	215 (41.3%)	305 (58.7%)	520 (100%)	0.126	1.34 (0.92–1.96) 1
	No	20 (30.8%)	45 (69.2%)	65 (100%)		
Total daily screen time	> 1 h	163 (39.3%)	252 (60.7%)	415 (100%)	0.977	1.00 (0.92–1.08) 1
	< 1 h	74 (39.2%)	115 (60.8%)	189 (100%)		
Prolonged screen use	> 4 h	42 (42.4%)	57 (57.6%)	99 (100%)	0.662	1.02 (0.92–1.14) 1
	< 4 h	195 (38.6%)	310 (61.4%)	505 (100%)		
Family structure	Non-nucleated	64 (48.1%)	69 (51.9%)	133 (100%)	0.013	1.31 (1.05–1.62) 1
	Nucleated	173 (36.7%)	298 (60.5%)	471 (100%)		
Caregiver’s education	< 8 years	51 (38.3%)	82 (61.7%)	133 (100%)	0.812	0.97 (0.76–1.23) 1
	> 8 years	186 (39.5%)	258 (60.5%)	471 (100%)		
Per capta income (R$)	≥ 551.00	108 (46.4%)	125 (53.6%)	233 (100%)	0.015	1.31 (1.05–1.63) 1
	≤ 550.00	84 (35.5%)	15 (64.7%)	238 (100%)		
Gender	Male	118 (42.0%)	163 (39.1%)	281 (100%)	0.206	1.13 (0.93–1.38) 1
	Female	119 (37.0%)	203 (63.0%)	322 (100%)		
**DISORDERS IN INITIATING AND MAINTAINING SLEEP**						
Use before bedtime	Yes	276 (53.1%)	244 (46.9%)	520 (100%)	0.030	1.43 (1.03–1.99) 1
	No	24 (36.9%)	41 (63.1%)	65 (100%)		
Total daily screen time	> 1 h	220 (53.0%)	195 (47.0%)	415 (100%)	0.141	1.07 (0.97–1.16) 1
	< 1 h	88 (46.6%)	101 (53.4%)	189 (100%)		
Prolonged screen use	> 4 h	55 (55.6%)	44 (44.4%)	99 (100%)	0.371	1.05 (0.94–1.17) 1
	< 4 h	253 (50.1%)	252 (49.9%)	505 (100%)		
Family structure	Non-nucleated	70 (52.6%)	63 (47.4%)	133 (100%)	0.665	1.04 (0.86–1.25) 1
	Nucleated	238 (50.5%)	233 (49.5%)	471 (100%)		
Caregiver’s education	< 8 years	61 (45.9%)	72 (54.1%)	133 (100%)	0.197	0.87 (0.71–1.07) 1
	> 8 years	247 (52.4%)	224 (47.6%)	471 (100%)		
Per capta income (R$)	≥ 551.00	142 (60.9%)	91 (39.1%)	233 (100%)	< 0.001	1.43 (1.19–1.72) 1
	≤ 550.00	101 (42.4%)	137 (57.6%)	238 (100%)		
Gender	Male	141 (50.2%)	112 (43.6%)	281 (100%)	0.680	0.96 (0.83–1.13) 1
	Female	167 (51.9%)	155 (48.1%)	322 (100%)		
**RESPIRATORY SLEEP DISORDERS**						
Use before bedtime	Yes	267 (51.3%)	253 (48.7%)	520 (100%)	0.448	1.11 (0.84–1.46) 1
	No	30 (46.2%)	35 (58.8%)	65 (100%)		
Total daily screen time	> 1 h	216 (52.0%)	199 (48.0%)	415 (100%)	0.135	1.07 (0.98–1.16) 1
	< 1 h	86 (45.5%)	103 (54.5%)	189 (100%)		
Prolonged screen use	> 4 h	55 (55.6%)	44 (44.4%)	99 (100%)	0.294	1.05 (0.95 1.18) 1
	< 4 h	247 (48.9%)	258 (51.1%)	505 (100%)		
Family structure	Non-nucleated	66 (49.6%)	67 (50.4%)	133 (100%)	0.922	0.99 (0.81–1.20) 1
	Nucleated	236 (50.1%)	235 (49.9%)	471 (100%)		
Caregiver’s education	< 8 years	55 (41.4%)	78 (58.6%)	133 (100%)	0.034	0.78 (0.63–0.98) 1
	> 8 years	247 (52.4%)	224 (47.6%)	471 (100%)		
Per capta income (R$)	≥ 551.00	121 (51.9%)	112 (48.1%)	233 (100%)	0.489	1.06 (0.89–1.27) 1
	≤ 550.00	116 (48.7%)	122 (51.3%)	238 (100%)		
Gender	Male	140 (49.8%)	141 (50.2%)	281 (100%)	0.905	0.990 (0.84–1.16) 1
	Female	162 (50.3%)	160 (49.7%)	322 (100%)		
**EXCESSIVE SLEEPNESS**						
Use before bedtime	Yes	161 (31.0%)	359 (69.0%)	520 (100%)	0.212	1.34 (0.84–2.13) 1
	No	15 (23.1%)	50 (76.9%)	65 (100%)		
Total daily screen time	> 1 h	143 (34.5%)	272 (65.5%)	415 (100%)	< 0.001	1.16 (1.07–1.24) 1
	< 1 h	37 (19.6%)	152 (80.4%)	189 (100%)		
Prolonged screen use	> 4 h	44 (44.4%)	55 (55.6%)	99 (100%)	0.001	1.18 (1.07–1.32) 1
	< 4 h	136 (26.9%)	369 (73.1%)	505 (100%)		
Family structure	Non-nucleated	40 (30.1%)	93 (69.9%)	133 (100%)	0.938	1.01 (0.75–1.35) 1
	Nucleated	140 (29.7%)	331 (70.3%)	471 (100%)		
Caregiver’s education	< 8 years	42 (31.6%)	91 (68.4%)	133 (100%)	0.609	1.07 (0.80–1.43) 1
	> 8 years	138 (29.3%)	333 (70.7%)	471 (100%)		
Per capta income (R$)	≥ 551.00	80 (34.3%)	153 (65.7%)	233 (100%)	0.214	1.18 (0.90–1.51) 1
	≤ 550.00	69 (29.0%)	110 (46.2%)	238 (100%)		
Gender	Male	90 (32.0%)	191 (68.0%)	281 (100%)	0.275	1.146 (0.897–1.463) 1
	Female	90 (28.0%)	231 (75.2%)	322 (100%)		
**AROUSAL DISORDERS**						
Use before bedtime	Yes	179 (34.4%)	341 (65.6%)	520 (100%)	0.877	0.97 (0.68–1.38) 1
	No	23 (35.4%)	42 (64.6%)	65 (100%)		
Total daily screen time	> 1 h	149 (35.9%)	266 (64.1%)	415 (100%)	0.094	1.07 (0.99–1.16) 1
	< 1 h	55 (29.1%)	134 (70.9%)	189 (100%)		
Prolonged screen use	> 4 h	45 (45.5%)	54 (55.5%)	99 (100%)	0.015	1.14 (1.03–1.27) 1
	< 4 h	159 (31.5%)	346 (68.5%)	505 (100%)		
Family structure	Non-nucleated	39 (29.3%)	94 (70.7%)	133 (100%)	0.231	0.83 (0.62–1.12) 1
	Nucleated	165 (35.0%)	306 (65.0%)	471 (100%)		
Caregiver’s education	< 8 years	35 (26.3%)	98 (73.7%)	133 (100%)	0.049	0.73 (0.53–0.99) 1
	> 8 years	169 (35.9%)	302 (64.1%)	471 (100%)		
Per capta income (R$)	≥ 551.00	84 (36.1%)	149 (63.9%)	233 (100%)	0.217	1.17 (0.91–1.51) 1
	≤ 550.00	73 (30.7%)	165 (69.3%)	238 (100%)		
Gender	Male	93 (33.1%)	188 (66.9%)	281 (100%)	0.722	0.960 (0.767–1.202) 1
	Female	111 (34.5%)	211 (65.5%)	322 (100%)		
**SLEEP-WAKE TRANSITION DISORDERS**						
Use before bedtime	Yes	286 (55.0%)	234 (45.0%)	520 (100%)	0.688	1.05 (0.82–1.34) 1
	No	34 (52.3%)	31 (47.7%)	65 (100%)		
Total daily screen time	> 1 h	221 (53.3%)	194 (46.7%)	415 (100%)	0.966	0.99 (0.91–1.08) 1
	< 1 h	101 (53.4%)	88 (46.6%)	189 (100%)		
Prolonged screen use	> 4 h	64 (64.6%)	35 (35.4%)	99 (100%)	0.026	1.12 (1.02–1.25) 1
	< 4 h	258 (51.1%)	247 (48.9%)	505 (100%)		
Family structure	Non-nucleated	80 (60.2%)	53 (39.8%)	133 (100%)	0.059	1.17 (0.99–1.37) 1
	Nucleated	242 (51.4%)	229 (48.6%)	471 (100%)		
Caregiver’s education	< 8 years	66 (49.6%)	67 (50.4%)	133 (100%)	0.348	0.91 (0.75–1.01) 1
	> 8 years	256 (54.5%)	215 (45.6%)	471 (100%)		
Per capta income (R$)	≥ 551.00	130 (55.8%)	103 (44.2%)	233 (100%)	0.371	1.08 (0.93–1.27) 1
	≤ 550.00	123 (51.7%)	115 (48.3%)	238 (100%)		
Gender	Male	148 (52.7%)	133 (47.3%)	281 (100%)	0.737	0.97 (0.83–1.13) 1
	Female	287 (52.3%)	262 (47.7%)	322 (100%)		

*SDSC* Sleep Disturbance Scale for Children *PR*_*c*_ crude prevalence ratio *CI* confidence interval

It was also observed that among children who took more than 60 min to fall asleep, 93.9% had exposure to screen use at bedtime (*p* = 0.021). Additionally, the media use at bedtime was significantly associated with caregivers who worked outside the home; 90.7% of these children had used media to fall asleep in the past 6 months (*p* = 0.036). Additional significant associations are detailed in Table 3.

In the multiple Poisson regression analysis, after adjusting for potential confounders, prolonged screen time exposure remained significantly associated with the domains of excessive daytime sleepiness (*p* = 0.015; PR_a_ = 1.16; 95% CI = 1.05–1.28), sleep–wake transition disorders (*p* = 0.026; PR_a_ = 1.12; 95% CI = 1.01–1.25), and the total SDSC score (*p* = 0.003; PR_a_ = 1.16; 95% CI = 1.05–1.28).

The association between media use at bedtime and disorders of initiating and maintaining sleep was no longer significant in the adjusted analysis. However, daily media use of more than 1 h was significantly associated with initiating and maintaining sleep disorders (*p* = 0.025; PR_a_ = 1.26; 95% CI = 1.03–1.55) and sleep hyperhidrosis (*p* = 0.046; PR_a_ = 1.12; 95% CI = 1.00–1.27). Adjusted prevalence ratios for other variables are present in Table 4.

**Table 4:** Multiple Poisson regression analysis of the study population (*N* = 604; ITAJAÍ-SC, BRAZIL- 2019)

Variables		PR_a_ (IC 95%)	***p*-value**
**SDSC TOTAL**			
Prolonged screen use	> 4 h	1.23 (1.06- 1.44)	0.006
	< 4 h	1	
Per capta income (R$)	≥ 551,00	1.23 (1.06 – 1.44)	0.005
	≤ 550,00	1	
HYPERHIDROSIS			
Family Structure	Non-nucleated	1.30 (1.02- 1.64)	0.029
	Nucleated	1	
Renda per capta	≥ 551,00	1,32 (1,06—1,64)	0.013
	≤ 550,00	1	
DISORDERS IN INITIATING AND MAINTAINING SLEEP			
Prolonged screen use	> 1 h	1.26 (1.03- 1.55)	0.025
	< 1 h	1	
Per capta income (R$)	≥ 551,00	1,41 (1,18- 1,69)	< 0.001
	≤ 550,00	1	
RESPIRATORY SLEEP DISORDERS			
Prolonged screen use	> 1 h	1,06 (0,979 -1,16)	0.143
	< 1 h	1	
Caregiver’s education	< 8 years	0,896 (0,815–0,985)	0.023
	> 8 years	1	
EXCESSIVE SLEEPINESS			
Prolonged screen use	> 4 h	1.18 (1.07–1.24)	0.001
	< 4 h	1	
AROUSAL DISORDERS			
Prolonged screen use	> 4 h	1.14 (1.02–1.27)	0.015
	< 4 h	1	
SLEEP–WAKE TRANSITION DISORDERS			
Prolonged screen use	> 4 h	1.12 (1.01–1.25)	0.026
	< 4 h	1	
Family Structure	Non-nucleated	1.09 (0.99—1.21)	0.059
	Nucleated	1	

*SDSC* Sleep Disturbance Scale for Children *PR*_*a*_ adjusted prevalence ratio *CI* confidence interval

## Discussion

The results of this study indicate that the electronic screen time exposure use among Brazilian preschoolers exceeds the recommendations established by the AAP and BSP (Hill et al. 2016; BSP, 2019). In the present study, 189 children (33.1%) used media for up to 1 h per day, whereas almost 18% of the children reported media use exceeding four hours daily—substantially surpassing the 1-h daily limit advised by both organizations [10, 11]. These findings are concerning, given that the established guidelines aim to limit daily usage due to the potential risks associated with prolonged media exposure on children’s quality of life, such as developmental changes, obesity risk, and sleep disturbances [10, 11]. Previously, excessive media use may negatively affect children’s development, health, and psychological status [6, 14, 23]. Furthermore, it has been associated with impairments in language, cognition, and social interaction [13, 15]. It may also reduce opportunities for interaction with caregivers and peers [10, 24].

Bedtime media use is strongly associated with poorer sleep quality and has been recognized as an important modifiable risk factor for sleep disturbances in children and adolescents [6, 9, 14, 23]. In thepresent study, nearly 90% (*n *= 520) of children reported using some form of electronic media to fallasleep, prevalence considerable higher than reported in previous study, 75% of American children andadolescents report having at least 1 screen media device in their bedroom, with roughly 60% reportingregular use of these device during the hour before beadtime [6, 9]. The potencial effects of screen usebefore bedtime on sleep quality could be mediated by multiple biological, pscychological, and behavioralfactors, due to observational and cross-sectional nature of the evidence, causality is not be determined.Evidence suggest as potencial mechanisms: the time displacement of sleep time, screen use could lead tobehavioral beadtime delay; psychological stimulation from media content, effects of light-emitting screensthat could influence on alerting, and circadian effects [6, 23, 25, 26].

The present study identified significant associations between prolonged daily media use (≥ 4 h) and/or media use at bedtime and the presence of sleep disorders in Brazilian preschoolers. These findings are consistent with previous studies reporting that electronic media use is linked to poorer sleep quality in children and adolescents [1, 5, 23, 27, 28, 29]. In a systematic review, screen time is adversely associated with sleep outcomes (primarily shortened duration and delayed timing) in 90% of studies [6].

Studies using the SDSC among preschool populations in Chile, Finland, and Italy have also reported associations between total daily screen time and excessive daytime sleepiness, sleep–wake transition disorders, awakening disorders, respiratory disorders, and the overall SDSC score, which are consistent with the findings of the present study [1, 3, 27]. A study conducted among American children using the Children’s Sleep Habits Questionnaire also reported similar findings, demonstrating that daytime media use is associated with poorer sleep quality and increased daytime fatigue in preschoolers [4].

Regarding our rates sleep disorders, the prevalence Sleep Breathing Disorders (SBD) observed in the present study was noteworthy. SBD including snoring and sleep-disordered breathing symptoms are recognized risk factors for sleep fragmentation, daytime sleepiness, behavioral problems, and impaired quality of life in children. Therefore, the relatively high prevalence of SBD found in our sample highlights the importance of early identification and monitoring of respiratory sleep symptoms in preschool-aged children. Furthermore, awakening disorders and sleep–wake transition disorders include issues such as nightmares, night terrors, sleep tremors, sleepwalking, and sleep talking, which are often associated with sleep agitation [3]. The association between these disorders and excessive screen exposure may be related not only to screen time but also to the nature of the content consumed. Interactive activities such as gaming, social media engagement, and online communication have been associated with greater sleep disruption than passive activities such as television viewing [6, 14, 26]. Exposure to adult or violent programming and certain video games has been previously associated with poorer sleep quality and heightened agitation [3, 26, 27].

Media use before bedtime was significantly associated with sleep hyperhidrosis and disorders related to initiating or maintaining sleep in univariate analysis. However, when other independent variables were considered, initiating and maintaining sleep disorders were associated with total exposure time rather than pre-sleep use specifically. Given the cross-sectional design of this study, longitudinal research is recommended to better understand and determine the real impact of pre-sleep media use, as poor sleep habits may evolve into sleep disorders if persistent. Additionally, it was found that approximately 90% of the children who required more than 60 min to fall asleep reported using media devices before bedtime. The literature suggests that exposure to bright light, especially blue-spectrum light emitted by screens, reduces drowsiness, promotes alertness, delays sleep onset, and shortens total sleep duration [28–30]. This increased alertness and resulting irregular sleep patterns may contribute to metabolic dysregulation and decreased melatonin levels, potentially triggering episodes of sleep hyperhidrosis [28–30]. Recent consensus recommendations from the National Sleep Foundation concluded that screen use before bedtime, particularly the content consumed, negatively affects sleep health in children and adolescents and supports behavioral strategies aimed at limiting media exposure before sleep [26].

Sleep disturbances are influenced by a complex interplay of family, behavioral, environmental, and socioeconomic factors that may operate through multiple pathways, as indirect path [31–34]. In the present study, socioeconomic characteristics and family structure were significantly associated with sleep outcomes. In addition to education, per capita income represents a key socioeconomic health indicator. Income and education presented collinearity (individuals with higher education typically have higher incomes); thus, this study also considered income as an influencing factor, as previously [35]. Interestingly, higher parental education levels were associated with a higher prevalence of sleep disturbances among children in the present study. Although lower socioeconomic status is generally associated with poorer health outcomes and reduced access to healthcare resources [32–34, 36], the relationship between socioeconomic indicators and children’s sleep remains inconsistent in the literature [34, 36]. While some studies have reported that lower parental education and family income are associated with increased sleep problems and poorer sleep quality [32–34, 36], linking lower socioeconomic factors with more likely to experience disruptive sleep conditions, e.g., shared bedroons, noisy environment, and temperature or bed discomfort [31, 34, 37]. Other investigations have found no association or even better sleep outcomes among children from families with lower educational attainment [1, 38, 39].

Several indirect mechanisms could link parental education and income with childhood sleep outcomes. Families with higher educational attainment often provide children with earlier and greater access to extracurricular activities, and academically demanding routines, factors that may increase exposure to behaviors associated with delayed bedtimes and insufficient sleep [33, 34, 38]. Additionally, highly educated parents may experience greater work-related demands, occupational stress, and time constraints, which can affect family routines and children’s sleep schedules. Higher academic expectations and increased participation in structured activities have also been associated with shorter sleep duration, particularly among school-aged children and adolescents. Furthermore, because sleep disturbances were assessed through parental reports, cultural, educational, and social factors may influence how parents perceive and report sleep-related symptoms in their children [40]. This reporting effect may partially explain the association observed in our study.

Therefore, the present findings should not be interpreted as suggesting that lower parental education is protective against sleep disturbances. Rather, they highlight the complex and multifactorial nature of the relationship between socioeconomic factors and children’s sleep. Future longitudinal studies incorporating objective sleep measures, family routines, parental stress, sleep hygiene practices, digital media use, and cultural factors are needed to clarify the mechanisms underlying these associations.

Regarding family structure, children from non-nuclear families showed a higher prevalence of sleep–wake transition disorders and sleep hyperhidrosis. Similar findings indicate that children in single-parent households are more likely to follow media use patterns, potentially leading to reduced sleep quality and an increased risk of developing sleep disorders [9, 10]. Although our analyses were adjusted for important sociodemographic variables, including household income, family structure, and parental education, we did not assess other factors known to influence children’s sleep, such as physical activity levels, parental sleep and screen-use behaviors, the presence of electronic devices in the bedroom, and family bedtime routines [25]. These factors may represent additional sources of residual confounding and could partially explain the associations observed between screen time and sleep disturbances. Future longitudinal studies incorporating a broader range of behavioral and family-context variables are warranted to better elucidate the independent contribution of screen exposure to children's sleep health.

Caution should be taken in generalizing these results and conclusions to other preschool populations, besides the methodological rigor of this study. It is important to account for the specific socioeconomic and demographic characteristics of the population studied, as these factors have a direct influence sleep disorder prevalence. Due to the cross-sectional nature of the study, the findings represent a single moment in time, which limits the ability to draw causal inferences. Another limitation is that screen time was assessed by children’s device use in the home environment without differentiation according to screen modality, content type, timing of exposure, or parental involvement. Previous research suggests that the relationship between screen use and sleep depends not only on the duration of exposure but also on the type of content being consumed [14, 25]. Interactive activities such as gaming, social media engagement, and online communication have been associated with greater sleep disruption than passive activities such as television viewing [14, 25]. Therefore, the observed associations should be interpreted as reflecting overall domestic screen exposure rather than specific patterns of screen use. Future research incorporating more comprehensive assessments may provide a more nuanced understanding of the relationship between screen exposure and sleep disturbances in childhood.

The results of this study support the hypothesis that exposure to screen of electronic devices, mainly prolonged daily media use and/or use before bedtime, is associated with higher prevalence of sleep disorders in the preschool population. These findings reinforce the need for pediatric guidance on limiting daily screen exposure and avoiding electronic media use before bedtime. It is imperative that healthcare professionals, particularly those who care children and their caregivers, offer parent education about bedtime routines, emphasizing the importance of responsible media use. In this context, the American Academy of Pediatrics, endorsing the recommendations of the American Academy of Sleep Medicine, advises against the presence of screen-based devices in children’s bedrooms and recommends discontinuing screen use at least 30 min before bedtime. The guidelines further highlight the importance of effective communication between healthcare professionals and parents regarding the impact of screen exposure on sleep quality and duration, as well as the promotion of healthy sleep habits from early childhood [26].

## Data Availability

No datasets were generated or analysed during the current study.
